# Firearm Storage and Firearm Suicide

**DOI:** 10.1001/jamanetworkopen.2025.19266

**Published:** 2025-07-07

**Authors:** Matthew Miller, Joeseph Wertz, Sonja A. Swanson, Joseph A. Simonetti, Yifan Zhang, Deborah R. Azrael

**Affiliations:** 1 Department of Public Health and Health Sciences, Bouvé College of Health Sciences, Northeastern University, Boston, Massachusetts; 2 Department of Surgery, University of California, Irvine; 3 Department of Epidemiology, University of Pittsburgh School of Public Health, Pittsburgh, Pennsylvania; 4 Firearm Injury Prevention Initiative, University of Colorado Anschutz School of Medicine, Aurora; 5 Department of Health Policy, Stanford University School of Medicine, Stanford, California; 6 Department of Health Policy and Management, Harvard T.H. Chan School of Public Health, Harvard Injury Control Research Center, Boston, Massachusetts

## Abstract

**Importance:**

Suicide-prevention interventions often recommend removing firearms from the homes of individuals at elevated risk of suicide or, short of removal, locking and unloading all household firearms. The recommendation to remove firearms is based on strong and consistent evidence. For adults, however, the recommendation to lock firearms is based on few studies with inconsistent findings.

**Objective:**

To assess the association between firearm storage practices and suicide method by sex and age.

**Design, Setting, and Participants:**

This case-control study investigated decedents aged 15 years or older who lived in a home with firearms in the last year of their life and who died by suicide. Data were from the 1993 National Mortality Followback Survey. Analyses were conducted from June 1, 2024, to March 30, 2025.

**Exposures:**

The presence of 1 or more unlocked firearm in the decedent’s residence and/or 1 or more loaded firearm.

**Main Outcomes and Measures:**

The main outcome was firearm storage practices by suicide method. Logistic models, including both exposures, compared the relative odds of exposure among decedents who died by firearm suicide vs nonfirearm suicide, adjusted for sex, age, and region of residence at the time of death.

**Results:**

Among the 725 individuals who died by suicide (mean [SD] age, 47.1 [19.7] years; 554 males [85.0%]), 606 (83.6%) decedents died by firearm suicide and 119 (16.4%) died by nonfirearm suicide. Adult suicide decedents who used firearms were neither more nor less likely than those who used other suicide methods to have lived in a home where all firearms were locked (odds ratio [OR], 1.15 [95% CI, 0.67-1.95]) or unloaded (OR, 0.78 [95% CI, 0.44-1.36]). Corresponding ORs for locked firearms were 1.29 (95% CI, 0.69-2.44) for men and 0.58 (95% CI, 0.24-1.41) for women; for unloaded firearms, ORs were 0.80 (95% CI, 0.41-1.56) for men and 0.61 (95% CI, 0.25-1.51) for women. Among adolescent and young adult (hereinafter adolescent) decedents aged 15 to 20 years, approximately half (26 of 43 [60.5%]) who died by firearm suicide, but none of the 7 who died by nonfirearm suicide, had lived in a home with unlocked firearms. Among adolescents in households in which all firearms were locked, suicide method was not associated with whether any firearm was unloaded (OR, 1.36 [95% CI, 0.10-18.9]).

**Conclusions and Relevance:**

In this case-control study, neither locking nor unloading household firearms was associated with whether adults used a firearm in their suicide. By contrast, adolescents who died by firearm suicide were far more likely to have lived in a household with unlocked firearms than were adolescent decedents who died by nonfirearm suicide methods. Suicide-prevention approaches that aim to reduce suicide mortality, especially for adult subpopulations likely to own firearms, should focus on firearm access rather than storage practices.

## Key Points

**Question:**

Is the suicide method used by people who die by suicide associated with how firearms in their homes were stored?

**Findings:**

In this case-control study of 725 decedents who died by suicide, the odds of dying by firearm suicide were substantially lower for adolescents and young adults (aged 15 to 20 years), but not for adults, when all firearms in their homes were locked.

**Meaning:**

The findings of this study suggest that to help prevent suicide among adults living in homes with firearms, interventions should focus on reducing firearm access, rather than on specific storage practices.

## Teaser

This case-control study assesses the association between firearm storage practices and suicide method (firearm vs nonfirearm) among individuals who lived in a home with firearms in the last year of their life and died by suicide.

## Introduction

Firearms play an outsized role in US suicide deaths.^1^ In response, medical associations seeking to prevent suicide by youths (eg, the American Academy of Pediatrics) and by adult gun owners (eg, the Veterans Health Administration and the US Department of Defense) recommend removing firearms from the home or, short of removal, locking and/or unloading all household firearms.^2,3,4,5,6,7^

The recommendation to remove firearms is based on strong and consistent evidence that the presence of firearms in a home substantially increases the likelihood that members of that household will die by suicide. Interventions that have reduced the availability of highly lethal methods of suicide, including firearms, have resulted in large declines in suicide rates by the restricted method (eg, firearms) without appreciable changes in suicide rates by other methods.^8,9,10,11,12,13,14^ Observational studies have also consistently found that living in a home with firearms increases the risk of firearm suicide without appreciably affecting the risk of suicide using other methods.^15,16,17,18,19,20,21,22,23,24,25,26,27^

The recommendation to lock and/or unload all household firearms, by contrast, is supported by fewer studies—and for adults, the findings are uncertain and inconsistent.^17,18,28,29,30,31^ Specifically, 3^18,28,29^ of 5 studies^17,18,28,29,31^ reported protective effect estimates of locking up household firearms on adult suicide mortality (2 studies^17,31^ report null estimates). Of these 3 studies, 1 reported estimates with wide 95% CIs that include the null,^18^ another focused on suicides among older adults,^28^ and the third estimated the effect of locking firearms on firearm suicide using a national sample of decedents that excluded anyone known to have expressed a wish to die in the last month of life.^29^ The 2 studies that report a null association between locking status and suicide focused on a national sample of decedents who died in their home^17^ and a sample of US Army personnel on active service duties.^31^

Of the 5 studies that examined the association between storage practices and adult suicide, only 2 (Shenassa et al^29^ and Dahlberg et al^17^) incorporated data about nonfirearm suicide (or any other negative control outcome) in assessing the association of storage with mortality.^32,33^ Both studies used data from the 1993 National Mortality Followback Survey (NMFS), and both report that decedents who died by firearm suicide were an order of magnitude more likely to live in homes with firearms than were decedents who died by nonfirearm suicide. Yet, the 2 studies draw starkly conflicting conclusions about the association of storage practices with suicide risk among suicide decedents in households with guns. Specifically, Dahlberg et al^17^ concluded that neither locking nor unloading household firearms mitigated the suicide risk posed by living in a household with firearms, whereas Shenassa et al^29^ concluded that “firearm owners who keep their firearms locked or unloaded were at least 60% less likely to die from firearm-related suicide than those who store their firearms unlocked and/or loaded”—a protective effect of in-home storage practices on firearm suicide nearly as large as that reported for youths in a community-based case-control study published the following year.^30^

The current study revisits the 1993 NMFS—the only publicly available source of information about firearm storage and suicide by method—to reconcile the contradictory inferences drawn by Shenassa et al^29^ and Dahlberg et al.^17^ We assume that if, when, and for whom locking and/or unloading household firearms prevents suicide, it does so by meaningfully restricting access to firearms (but not access to other suicide methods). As such, insofar as these storage practices are effective barriers to firearms access, we assume that these storage practices will be less common among suicide decedents who used firearms than among suicide decedents who used nonfirearm methods. Thus, we considered nonfirearm suicide a useful negative control outcome for assessing the association between storage and suicide.^33,34^ Because routine in-home storage practices may plausibly pose a more formidable access barrier for nonowners than for the gun owners with whom they live and given that firearm ownership is more common among males than females and among adults than adolescents,^23,35,36,37,38^ we stratified our analyses by sex and age (adolescents and young adults aged 15 to 20 years [hereinafter adolescents] vs adults).

## Methods

### Study Sample

In this case-control study, data were analyzed from the 1993 NMFS, a survey based on a representative 10% sample of decedents aged 15 years or older who died in 1993.^39^ Data are representative of individuals aged 15 years or older who resided and died in the US, excluding South Dakota, from January 1 through December 31, 1993.

Information about decedents came from death certificates, coroner or medical examiner records, and interviews with people familiar with the decedent (hereinafter informants). The decedent’s next of kin, identified on the death certificate as having provided information to the funeral director, were initially contacted by letter and asked to participate. When an informant was not identified this way or could not be located, another person familiar with the decedent was recruited by letter. Informant interviews, conducted approximately 6 months after the date of death, included questions about firearms in the home of the decedent in the last year of life (but not about who in the household owned firearms). Among identified informants, 83% participated in the survey. Analyses involved only decedents who died by suicide and had lived in a household with firearms in their last year of life.

Owing to the use of existing deidentified data, this study was exempt from institutional review board approval and the need for informed consent according to the Common Rule. We followed the Strengthening the Reporting of Observational Studies in Epidemiology (STROBE) reporting guideline.

### Measures

#### Outcomes

We compared storage practices among decedents who died by firearm suicide with storage practices among decedents who died by nonfirearm suicide. Decedents who died by suicide were identified using the NMFS-abstracted death certificate manner of death and further characterized as either firearm or nonfirearm suicides according to the external cause of the *International Classification of Diseases, Ninth Revision, Clinical Modification* injury codes noted on their death certificates, which were linked to survey-based data obtained from next of kin about the decedent (firearm suicide: E955.0-E955.4; nonfirearm suicide: E950.x-954.x and E956.x-958.x).

#### Exposures

The key exposures were the presence of at least 1 unlocked firearm in the decedent’s residence and the presence of at least 1 loaded firearm in their residence. Unless decedents lived in a home with only 1 firearm, the NMFS’ skip logic did not allow us to determine whether any guns were both loaded and unlocked. For decedents with a single firearm, we considered the firearm locked if the respondent indicated that it was kept in a locked place (such as a drawer, cabinet, or closet) or stored elsewhere with a trigger lock or other locking mechanism. We considered the firearm unloaded if the informant indicated such or, following the study by Shenassa et al,^29^ if they indicated the firearm was taken apart. For decedents who lived in a household with more than 1 firearm, we considered the decedent to have lived in a home with only locked firearms if the informant indicated that all firearms were stored in a locked place, or if firearms were not said to have all been stored in a locked place, the only descriptor selected among storage options was “with a trigger lock or other locking mechanism” (vs “taken apart,” “assembled without a locking mechanism,” or “some other way”). Similar to those who were exposed to a single firearm, we classified those living with multiple firearms as not exposed to a loaded firearm only if the informant indicated that all firearms were stored taken apart or that all firearms stored intact were said to have been unloaded.

#### Covariates

The decedent’s age, sex (female or male), and region of residence at the time of death (Northeast, Midwest, South, or West), as ascertained from the death certificate, were included in our primary analyses. Additional sociodemographic and behavioral characteristics adjusted for in the studies by Dahlberg et al^17^ and Shenassa et al^29^ were included in sensitivity analyses. These additional characteristics were obtained from the questionnaire and included educational level (<4 years of high school, 4 years of high school, and at least some college), race and ethnicity (non-Hispanic Black, Hispanic, and non-Hispanic White or other [including American Indian or Alaska Native, Asian, Native Hawaiian or Other Pacific Islander, and unknown race or ethnicity]), and several categorical variables reflecting the frequency with which the decedent attended religious activities, engaged in moderate to vigorous physical activity, and had contact with family or friends in the last year of life. Other covariates included past-year alcohol consumption and whether the decedent experienced a demotion, job loss, quit a job, or retired; avoided needed health care; or saw a mental health professional. Categorical variables reflecting the number of depressive symptoms during the last month of life were also included (none, 1 to 2, 3 to 4, or 5 or more).

### Statistical Analysis

We compared storage practices among decedents who died by firearm suicide with storage practices among decedents who died by other suicide methods. Logistic regression adjusted simultaneously for locked and loaded status, as well as for decedent age, sex, and region of residence. Odds ratios (ORs) estimated the relative odds of suicide death by firearm compared with by nonfirearm methods if the decedent lived in a household in which all firearms were locked, adjusting for loaded status, and in which all firearms were unloaded, adjusting for locking status. Analyses were weighted to account for study design and survey nonresponse using the svy suite commands in Stata, version 16 (StataCorp LLC). Data analyses were conducted from June 1, 2024, to March 30, 2025.

Point estimates with 95% CIs less than 1 (eg, for locking) mean that locking all firearms was less common among firearm suicides than among nonfirearm suicides, consistent with locking all guns exerting a protective effect against firearm suicide. Point estimates with 95% CIs more than 1 suggest that locking household guns makes firearm suicide more likely. Estimates at or close to 1 suggest that locking all firearms was not associated with whether firearms or another method were used.

As in prior work using the NMFS, our primary analyses focused on decedents for whom storage practices were known.^17,29^ We sought to minimize misclassification error by restricting analyses to decedents for whom information about firearm storage practices were reported by close relatives (spouse, parent, or child) or by someone who had lived with the decedent in the last year of the decedent’s life.

We report several sensitivity analyses, including multiple imputations for firearm storage practices when informants reported that they knew a decedent’s household contained firearms but not how those firearms were stored. The imputation process used logistic regression models with covariates that prior literature suggests are factors in firearms storage decisions: presence of children in the home, race and ethnicity, age, sex, number of household firearms, region of residence, marital status, and educational level. We generated 50 imputed datasets; regression coefficients and SEs were estimated within each. Results from each dataset were pooled to create a single set of coefficients and SEs that accounted for the uncertainty introduced by the imputation process. Additional sensitivity analyses recoded “don’t know” responses to storage questions so as to produce estimates that minimized (and, separately, maximized) the protective effect of the association between locking and unloading firearms and firearm suicide. Finally, we compared estimates from our parsimonious multivariable model with estimates from models that adjusted for the covariates used in prior NMFS studies.

## Results

The study included 725 individuals who died by suicide (mean [SD] age, 47.1 [19.7] years; 171 females [15.1%] and 554 males [85.0%]). Of the total cohort, 45 (4.4%) were non-Hispanic Black, 30 (4.5%) were Hispanic, and 668 (88.0%) were non-Hispanic White or of other race or ethnicity.

Among the 725 decedents, 606 (83.6%) died by firearm suicide and 119 (16.4%) died by nonfirearm suicide. Compared with decedents who died by nonfirearm suicide, those who died by firearm suicide were more likely to be male (n = 75 [75.0%; 95% CI, 67.6%-82.4%] vs n = 479 [86.8%; 95% CI, 84.5%-89.1%], respectively) and to reside in the South (n = 33 [30.7%; 95% CI, 21.2-40.2] vs n = 273 [46.3%; 95% CI, 42.0-50.6], respectively) (Table 1). Overall, storage practices did not distinguish decedents who died by firearm suicide from those who died by nonfirearm suicide. However, differences in storage by suicide method became apparent when data were stratified by age and sex (eTable 1 in Supplement 1). For example, although men who died by firearm suicide and those who died by nonfirearm suicide methods lived in households in which firearms were stored similarly, a larger proportion of women who died by firearm suicide lived in homes in which at least 1 gun was unlocked (72 of 104 [69.2%] with known locked status) compared with women who died by nonfirearm suicide (24 of 39 [61.5%] with known locked status). Among adolescent decedents who died by suicide, only those who died by firearms lived in homes with unlocked guns.

**Table 1.** Characteristics of the Study Sample of Decedents Who Died by Suicide Living in Households With Firearms in the Last Year of Life^a^

Characteristic	Decedents who died by suicide	
	With firearm (n = 606)	Without firearm (n = 119)
Sex		
Female	127 (13.2) [10.9-15.5]	44 (25.0) [17.6-32.4]
Male	479 (86.8) [84.5-89.1]	75 (75.0) [67.6-82.4]
Age, mean (SD), y		
15-20	48 (7.5) [5.4-9.6]	8 (5.7) [1.7-9.7]
21-34	144 (24.5) [20.1-28.2]	29 (26.7) [17.9-35.7]
35-54	162 (32.2) [28.0-36.5]	36 (36.4) [26.4-46.3]
55-74	158 (24.7) [21.2-28.3]	30 (21.3) [13.8-28.8]
≥75	93 (11.1) [8.8-13.3]	16 (9.9) [4.8-14.8]
Race and ethnicity		
Hispanic	27 (5.1) [3.1-7.1]	3 (2.1) [0-4.6]
Non-Hispanic Black	38 (4.9) [2.7-6.7]	7 (5.0) [1.2-8.8]
Non-Hispanic White or other^b^	525 (90.4) [87.9-92.8]	104 (92.9) [88.4-97.5]
Missing	16 (2.6) [1.4-3.9]	5 (4.2) [0.1-7.9]
US region of residence		
Northeast	63 (12.5) [9.4-15.5]	12 (12.4) [5.5-19.2]
Midwest	129 (21.0) [17.6-24.4]	44 (36.0) [26.6-45.4]
South	273 (46.3) [42.0-50.6]	33 (30.7) [21.2-40.2]
West	141 (20.2) [17.0-23.5]	30 (20.9) [13.5-28.4]
Firearm storage		
Locked vs unlocked		
≥1 Unlocked	282 (44.7) [40.4-48.9]	56 (46.9) [37.0-56.8]
All locked	206 (35.6) [31.4-39.7]	47 (38.8) [29.2-48.3]
Don’t know	118 (19.7) [16.3-23.2]	16 (14.3) [7.2-21.5]
Loaded vs unloaded		
≥1 Loaded	208 (33.6) [29.5-37.6]	36 (31.9) [22.5-41.3]
All unloaded	295 (49.2) [45.0-53.5]	71 (58.5) [48.7-68.3]
Don’t know	103 (17.2) [14.0-20.5]	12 (9.6) [3.8-15.3]

^a^ Weighted distribution for decedents who died by suicide by suicide method. Data are presented as No. (%) [95% CI] unless otherwise indicated.

^b^ The National Mortality Followback Survey combined non-Hispanic White and other races and ethnicities (including American Indian or Alaska Native, Asian, Native Hawaiian or Other Pacific Islander, and unknown race or ethnicity) because the latter category comprised less than 0.5% of the study sample.

Adjusted estimates (Table 2) mirror the unadjusted association between the aforementioned firearm storage and suicide methods. For example, among adult decedents who died by suicide, most of whom were men, those who died by firearm suicide were neither more nor less likely than those who used nonfirearm suicide methods to have lived in a home in which all firearms were locked (OR, 1.15 [95% CI, 0.67-1.95]) or unloaded (OR, 0.78 [95% CI, 0.44-1.36]). A similar pattern was observed among men; those who died by firearm suicide were neither more nor less likely than men who died by nonfirearm suicide to have lived in a home in which all firearms were locked (OR, 1.29 [95% CI, 0.69-2.44]) or unloaded (OR, 0.80 [95% CI, 0.41-1.56]). For women, corresponding estimates were further from the null (in a protective direction), but with wide CIs, and similar for locked firearms (OR, 0.58 [95% CI, 0.24-1.41]) and for unloaded firearms (OR, 0.61 [95% CI, 0.25-1.51]). As noted, among adolescent decedents who died by suicide, only those who died by firearms lived in homes with unlocked guns. Among the 24 adolescents who died by suicide in a household in which all firearms were locked, the odds of dying by firearm suicide were not lower than the odds of dying by nonfirearm suicide when all firearms were also unloaded, albeit the corresponding point estimate has a wide 95% CI (OR, 1.36 [95% CI, 0.10-18.9]). Point estimates from multiply imputed analyses and from sensitivity analyses that included covariates used in prior studies were similar to those in our primary analyses (eTable 2 in Supplement 1).

**Table 2.** Association Between Storage Practices and Suicide Method (Firearm vs Nonfirearm) by Sex and Age Among Decedents Who Died by Suicide Living in Households With Firearms^a^

Analytic sample	No.	Odds ratio (95% CI)	
		All firearms locked^b^	All firearms unloaded^c^
All ages (both sexes)	547	0.94 (0.57-1.56)	0.81 (0.47-1.40)
All adults (both sexes)	497	1.15 (0.67-1.95)	0.78 (0.44-1.36)
Men	367	1.29 (0.69-2.44)	0.80 (0.41-1.56)
Women	130	0.58 (0.24-1.41)	0.61 (0.25-1.51)
Adolescents^d^	50	NA^e^	1.36 (0.10-18.9)^f^

Abbreviation: NA, not applicable.

^a^ Analyses were adjusted for locked and loaded storage practices in the same model, as well as age, sex, and US region of residence at the time of death (for adults). Analyses of adolescents were adjusted for age as a continuous variable, sex, and region of residence at the time of death. All analyses ignored records with missing information on storage practice.

^b^ Relative odds of all household firearms being locked among decedents who died by firearm vs nonfirearm suicides.

^c^ Relative odds of all household firearms being unloaded among decedents who died by firearm vs nonfirearm suicides.

^d^ Aged 15 to 20 years.

^e^ All decedents (7 of 7) who died by nonfirearm suicide lived in households in which all firearms were locked; more than half of those who died by firearm suicide (26 of 43 [60.5%]) lived in a household with unlocked firearms.

^f^ Because no adolescent who died by suicide using a firearm method had lived in a home with unlocked firearms, loading status was assessed among the 24 adolescent decedents who died by suicide who lived in households in which all guns were locked.

## Discussion

Our findings suggest that among decedents who died by suicide and lived in households with firearms, locking all firearms was associated with significantly reduced odds that an adolescent died by firearm suicide, consistent with the only case-control study to date to examine the association between firearm storage and adolescent suicide.^30^ For men, however, we found that neither locking nor unloading firearms was associated with reduced odds of firearm suicide. Estimates for women fell between those for men and those for adolescents, but with wide 95% CIs. The simplest explanation for why locking up household firearms would be inversely associated with firearm suicide among adolescent decedents who died by suicide but not among adult decedents who died by suicide is that adults who live in homes with guns are more likely than their children to have the key or combination to firearm storage devices in their home. The simplest explanation for why our point estimates for women fell between those for men and those for adolescents is that men are more likely to own firearms and thus, on average, are more likely to have greater control over and easier access to firearms in their home.^37,40^

Our finding that storage practices among adult decedents who died by suicide, most of whom were men, were not associated with the method used in their suicide is consistent with the null results reported by both Dahlberg et al^17^ and Shenassa et al^29^ for this same comparison in their analyses of the NMFS. Dahlberg et al^17^ interpreted their null results (eg, for locking all household firearms: OR, 1.2 [95% CI, 0.7-2.2]) as do we; among adults, locking all household guns did not appreciably affect the odds of suicide by firearms. The study by Shenassa et al,^29^ on the other hand, largely ignored their own null finding for this same comparison (OR, 0.87 [95% CI, 0.29-2.64]). Instead, their study focuses on comparisons of storage practices among decedents who died by firearm suicide with storage among decedents who died by natural causes. These comparisons, which produced ORs below 0.40 (with 95% CIs <1), are the basis for their study’s overarching conclusion that “firearm owners who keep their firearms locked or unloaded were at least 60% less likely to die from firearm related suicide than those who store their firearms unlocked and/or loaded.” However, given the study’s aforementioned null result, it follows that had storage among decedents who died by nonfirearm suicide been compared with storage among decedents who died by natural causes, it would have produced ORs similar in magnitude and direction to the “protective” estimates reported for decedents who died by firearm suicide. Unfortunately, policymakers and researchers continue to cite the study by Shenassa et al^29^ as empirical evidence that locking and unloading household firearms may substantially reduce suicide rates among adult populations, including populations consisting, in large part, of adult gun owners.^2,3,4,31,41,42,43,44,45^

### Limitations

Several limitations should be considered when interpreting our results. First, as with all studies using the NMFS, our measure of storage practices was based on information from interviews with people who knew the decedent but may not have had accurate knowledge about how firearms were stored. Second, it is possible that our findings are confounded by factors that we were unable to adjust for in analysis. A related concern is that our study involved only decedents. Deceased controls can introduce bias to the extent that the distribution of key exposures and confounders among controls is not representative of the population that gave rise to the cases. This concern is mitigated in the context of our study in that we adjusted for sex and age, which, in addition to method availability, are among the few well-established determinants of suicide method choice.^46,47,48,49,50,51,52^ Perhaps for this reason, point estimates from our parsimonious model were not meaningfully changed by including the covariates used by either Dahlberg et al^17^ or Shenassa et al.^29^ Finally, the association between storage and access may be different today than it was in 1993. As such, our findings should be considered in light of recent evidence that nearly one-quarter of US adolescents who reside in homes in which all firearms are locked can gain access to a loaded household gun in less than 5 minutes and that, contrary to their child’s report, most parents believe that their child could not gain access to a household firearm without help from an adult.^38^

## Conclusions

Suicide-prevention approaches that aim to meaningfully reduce mortality among adult gun owners currently encourage those at heightened risk of suicide to reduce access to firearms; many suggest that an effective way to do so is to lock up and unload household firearms.^5,6^ In this case-control study, firearm storage practices were associated with the suicide method used by adolescents but not by adults, underscoring the importance for interventions to focus on reducing firearm access rather than on adopting particular storage practices.

## Notes

Supplement 1. **eTable 1. **Storage of Firearms in the Last Year of Life for Decedents Who Lived in Households With Firearms and Died by Suicide, by Suicide Method, for Men, Women, and Adolescents 15 to 20 Years of Age, Weighted Proportion With Indicated Status (95% CI) **eTable 2. **Sensitivity Analyses: Association Between Firearm Storage Practices and Firearm Suicide Among Decedents Who Died by Suicide and Lived in Households With Firearms, by Sex and Age

Supplement 2.
Data Sharing Statement
