# Supplementary material for: Firearm Storage and Firearm Suicide
Source: JAMA Netw Open. 2025 Jul 7;8(7):e2519266. doi: 10.1001/jamanetworkopen.2025.19266 (PMC12235496; doi:10.1001/jamanetworkopen.2025.19266)
Supplement: Supplement 1. — eTable 1. Storage of Firearms in the Last Year of Life for Decedents Who Lived in Households With Firearms and Died by Suicide, by Suicide Method, for Men, Women, and Adolescents 15 to 20 Years of Age, Weighted Proportion With Indicated Status (95% CI) eTable 2. Sensitivity Analyses: Association Between Firearm Storage Practices and Firearm Suicide Among Decedents Who Died by Suicide and Lived in Households With Firearms, by Sex and Age [file jamanetwopen-e2519266-s001.pdf]

Supplemental Online Content

Miller M, Wertz J, Swanson SA, Simonetti JA, Zhang Y, Azrael DR. Firearm storage and firearm suicide. *JAMA Netw Open*. 2025;8(7):e2519266.
doi:10.1001/jamanetworkopen.2025.19266

eTable 1. Storage of Firearms in the Last Year of Life for Decedents Who Lived in Households With Firearms and Died by Suicide, by Suicide Method, for Men, Women, and Adolescents 15 to 20 Years of Age, Weighted Proportion With Indicated Status (95% CI)

eTable 2. Sensitivity Analyses: Association Between Firearm Storage Practices and Firearm Suicide Among Decedents Who Died by Suicide and Lived in Households With Firearms, by Sex and Age

This supplemental material has been provided by the authors to give readers additional information about their work.

© 2025 Miller M et al. *JAMA Network Open*.

**eTable 1.** Storage of Firearms in the Last Year of Life for Decedents Who Lived in Households With Firearms and Died by Suicide, by Suicide Method, for Men, Women, and Adolescents 15 to 20 Years of Age, Weighted Proportion With Indicated Status (95% CI)			
Analytic Sample	Firearm Storage Status*	Distribution of Firearm Storage Among Firearm Suicides	Distribution of Firearm Storage Among Non-Firearm Suicides
All adults (N=668: 557 Firearm and 111 Nonfirearm suicides)	≥1 Firearm Unlocked	44.1% (39.7-48.5)	49.7% (39.4-60.0)
	All Firearms Locked	35.5% (31.2-39.9)	35.6% (25.8-45.3)
	Don't Know Locked Status	20.4% (16.8-24.0)	14.7% (7.2-22.2)
Men (N=506: 438 Firearm and 68 Nonfirearm suicides)	≥1 Firearm Unlocked	41.6% (36.7-46.5)	47.4% (34.5-60.2)
	All Firearms Locked	36.8% (32.0-41.7)	35.8% (23.6-48.0)
	Don't Know Locked Status	21.6% (17.6-25.6)	16.8% (7.1-26.5)
Women (N=162: 119 Firearm and 43 Nonfirearm suicides)	≥1 Firearm Unlocked	60.2%(51.2-69.3)	56.3%(40.4-72.3)
	All Firearms Locked	27.1%(18.9-35.3)	34.9%(19.5-50.4)
	Don't Know Locked Status	12.6%(6.4-18.9)	8.7%(0.1-17.3)
Adolescents (N=57: 49 Firearm and 8 Nonfirearm Suicides)	≥1 Firearm Unlocked	52.1% (37.1-67.0)	0.0% (NA)
	All Firearms Locked	36.0%(21.6-50.3)	91.5% (70.4-100)
	Don't Know Locked Status	11.9% (1.7-22.2)	8.5% (0.0- 29.6)
All adults (N=668: 557 Firearm and 111 Nonfirearm suicides)	≥1 Firearm Loaded	34.0%(29.8-38.2)	32.9%(23.1-42.6)
	All Firearms Unloaded	47.7%(43.2-52.2)	57.5%(47.3-67.7)
	Don't Know Loaded Status	18.3%(14.8-21.7)	9.6%(3.6-15.6)
Men (N=506: 438 Firearm and 68 Nonfirearm suicides)	≥1 Firearm Loaded	31.7%(27.1-36.4)	32.1%(19.8-44.3)
	All Firearms Unloaded	49.4%(44.4-54.4)	57.9%(45.2-70.7)

© 2025 Miller M et al. *JAMA Network Open*.

	Don't Know Loaded Status	18.9%(15.0-22.7)	10.0%(2.3-17.6)
Women (N=162: 119 Firearm and 43 Nonfirearm suicides)	≥1 Firearm Loaded	Appendix 1	35.1%(19.7-50.5)
	All Firearms Unloaded	36.7%(27.8-45.7)	56.3%(40.3-72.2)
	Don't Know Loaded Status	14.5%(8.0-20.9)	8.6%(0.1-17.1)
Adolescents (N=57: 49 Firearm and 8 Nonfirearm Suicides)	≥1 Firearm Loaded	27.7% (14.0-41.3)	16.4%(0.0-53.5)
	All Firearms Unloaded	68.1%(53.9-82.2)	75.1%(34.9-100.0)
	Don't Know Loaded Status	4.2%(0.0-10.2)	8.5%(0.0-29.6)

© 2025 Miller M et al. *JAMA Network Open*.

eTable 2. Sensitivity Analyses: Association Between Firearm Storage Practices and Firearm Suicide Among Decedents Who Died by Suicide and Lived in Households With Firearms, by Sex and Age

Analytic Samples	All Locked Odds Ratio [95% CI]	All Unloaded Odds Ratio [95% CI]
Storage Status multiply imputed when storage missing		
All ages, both sexes (n=722)	All Locked: 1.05 [0.64-1.72]	All Unloaded: 0.90 [0.54-1.49]
All adults (both sexes) (n=665)	All Locked: 1.25 [0.74-2.11]	All Unloaded: 0.89 [0.53-1.52]
Men (n=503)	All Locked: 1.39 [0.75-2.57]	All Unloaded: 0.94 [0.50-1.76]
Women (n=162)	All Locked: 0.62 [0.26-1.50]	All Unloaded: 0.66 [0.27-1.63]
Adolescents 15-20 years of age (n=57)	All nonfirearm suicide decedents lived in homes where all firearms were locked	All Unloaded: 1.36 [0.10-18.9]^
Maximizes the protective effect of "safer storage"		
All ages, both sexes (n=610)	All Locked: 0.59 [0.36-0.97]	All Unloaded: 0.60 [0.33-1.01]
All adults (n=556/540)	All Locked: 0.69 [0.41-1.14]	All Unloaded: 0.57 [0.33-0.98]
Males (n=416/397)	All Locked: 0.72 [0.40-1.31]	All Unloaded: 0.58 [0.30-1.12]
Women (n=121/143)	All Locked: 0.48 [0.20-1.15]	All Unloaded: 0.46 [0.19-1.08]
Adolescents 15-20 (n=57)	All nonfirearm suicide decedents lived in homes where all firearms were locked	All Unloaded: 1.36 [0.10-118.9]^
Maximizes the protective effect of "unsafe" storage		
All ages, both sexes (n=610/591)	All Locked: 1.51 [0.94-2.42]	All Unloaded: 1.11 [0.67-1.85]
All adults (n=558/540)	All Locked: 1.96 [1.18-3.22]	All Unloaded: 1.09 [0.64-1.83]
Males (n=418/397)	All Locked: 2.25 [1.24-4.04]	All Unloaded: 1.10 [0.59-2.05]
Women (n=140/143)	All Locked: 0.81 [0.35-1.92]	All Unloaded: 0.87[0.36-2.11]
Adolescents 15-20 (n=57)	All Locked 0.23 [0.03-1.89]^^	NA
Adjusting for all covariates in Dahlberg et al (2004)*		
All adults (481)	All Locked: 1.05 [0.60-1.83]	All Unloaded: 0.77 [0.42-1.42]

© 2025 Miller M et al. *JAMA Network Open*.

Men (n=354)	All Locked: 1.22 [0.63-2.35]	All Unloaded: 0.85 [0.41-1.77]
Women (n=127)	All Locked: 0.50 [0.19-1.40]	All Unloaded 0.54 [0.21-1.40]
Adjusting for all covariates in Shenassa et al (2004)**		
All adults (372)	All Locked: 1.16 [0.61-2.21]	All Unloaded: 0.77 [0.38-1.54]
Men (n=255)	All Locked: 1.65 [0.74-3.67]	All Unloaded: 0.83 [0.33-2.10]
Women (n=109)	All Locked: 0.33 [0.07-1.66]	All Unloaded 0.99 [0.22-4.49]

^Because locking status predicted firearm suicide semideterministically for adolescents, analyses of the association between loaded firearms and firearm suicide were assessed among the 24 households with adolescents who lived in households where all firearms were locked. Because loaded status is never "don't know" among adolescents when locking status was reported to be known, the point estimates for loaded status conditioned on all guns being locked does not vary in imputed analyses verses analyses that maximize the "protective effect" of unloading all firearms.

Maximum protective effect estimates for "safer" storage are derived by assigning "don't know" responses to storage questions such that models produces point estimates that maximally exaggerate the protective effect of locking and of unloading all guns (i.e., all don’t knows for firearm suicides were coded as unlocked and unloaded). Maximum protective effect estimates for "unsafe" storage are derived by assigning "don't know" responses to storage questions such that models t maximally exaggerate the protective effect of "unsafe" storage (i.e., of having loaded firearms, of having unlocked firearms). The point estimates for maximized and minimized protective effect of locked status come from models that condition on loaded status being known, and vice-versa. ^^And allowed for an upper bound estimate of locking effect on adolescent firearm suicide.

* Covariates in Dahlberg 2004: sex, age group, race/ethnicity (on death certificate), education, marital status, residential status, and region of death.

** Covariates in Shenassa: sex, age, gender, race/ethnicity (on death certificate), education, region of residence, frequency of religious activities, physical activities, and contact with family/friends, alcohol consumption, employment change, avoidance/refusal of health care, depressive symptoms, and visits to a mental health professional. These covariates do not all pertain to adolescents and for this reason these sensitivity analyses are confined to adults.

© 2025 Miller M et al. *JAMA Network Open*.
