# Supplementary material for: Firearm Storage and Firearm Suicide
Source: JAMA Netw Open. 2025 Jul 7;8(7):e2519266. doi: 10.1001/jamanetworkopen.2025.19266 (PMC12235496; doi:10.1001/jamanetworkopen.2025.19266)
Supplement: Supplement 2. — Data Sharing Statement [file jamanetwopen-e2519266-s002.pdf]

Data Sharing Statement

Miller. Firearm Storage and Firearm Suicide. *JAMA Netw Open*. Published July 07, 2025. doi:10.1001/jamanetworkopen.2025.19266

Data
Data available: Yes
Data types: Deidentified participant data
How to access data: Data are publically available. We provide citations to the publically available data.
When available: With publication

Supporting Documents
Document types: None

Additional Information
Who can access the data: Anyone via the publically available website
Types of analyses: Any
Mechanisms of data availability: These data re publically available. We provide a citation and link to the repository
